# CD47 Expression in Circulating Tumor Cells and Circulating Tumor Microemboli from Non-Small Cell Lung Cancer Patients Is a Poor Prognosis Factor

**DOI:** 10.3390/ijms241511958

**Published:** 2023-07-26

**Authors:** Jacqueline Aparecida Torres, Angelo Borsarelli Carvalho Brito, Virgilio Souza e Silva, Iara Monique Messias, Alexcia Camila Braun, Anna Paula Carreta Ruano, Marcilei E. C. Buim, Dirce Maria Carraro, Ludmilla Thomé Domingos Chinen

**Affiliations:** 1 International Research Center, A.C. Camargo Cancer Center, São Paulo 01508-010, Brazil; jacque_a_torres@hotmail.com (J.A.T.);; 2 Department of Clinical Oncology, A.C. Camargo Cancer Center, São Paulo 01509-900, Brazil; 3 Faculdade de Medicina de Marília, São Paulo 17519-030, Brazil; 4 Translational Medicine Laboratory, Núcleo de Pesquisa e Ensino da Rede São Camilo, São Paulo 04014-002, Brazil

**Keywords:** lung cancer, non-small cell lung cancer (NSCLC), circulating tumor cells, circulating tumor microemboli, liquid biopsy, CD47

## Abstract

Circulating tumor cells (CTCs) and/or circulating tumor microemboli (CTM) from non-small cell lung cancer (NSCLC) patients may be a non-invasive tool for prognosis, acting as liquid biopsy. CTCs interact with platelets through the transforming growth factor-β/transforming growth factor-β receptor type 1 (TGF-β/TGFβRI) forming clusters. CTCs also may express the Cluster of Differentiation 47 (CD47) protein, responsible for the inhibition of phagocytosis, the “don’t eat me” signal to macrophages. Objectives: To isolate, quantify and analyze CTCs/CTMs from metastatic NSCLC patients, identify TGFβRI/CD47 expression in CTCs/CTMs, and correlate with progression-free survival (PFS). Methods: Blood (10 mL) was collected at two time-points: T1 (before the beginning of any line of treatment; T2 (60 days after initial collection). CTCs were isolated using ISET^®^. Immunocytochemistry was conducted to evaluate TGFβRI/CD47 expression. Results: 45 patients were evaluated. CTCs were observed in 82.2% of patients at T1 (median: 1 CTC/mL; range: 0.33–11.33 CTCs/mL) and 94.5% at T2 (median: 1.33 CTC/mL; 0.33–9.67). CTMs were observed in 24.5% of patients and significantly associated with poor PFS (10 months vs. 17 months for those without clusters; *p* = 0.05) and disease progression (*p* = 0.017). CTMs CD47+ resulted in poor PFS (*p* = 0.041). TGFβRI expression in CTCs/CTMs was not associated with PFS. Conclusion: In this study, we observed that CTC/CTM from NSCLC patients express the immune evasion markers TGFβRI/CD47. The presence of CTMs CD47+ is associated with poor PFS. This was the first study to investigate CD47 expression in CTCs/CTM of patients with NSCLC and its association with poor PFS.

## 1. Introduction

Lung cancer (LC) is a serious public health problem worldwide. In 2020, it was the second most frequent cancer and the most lethal solid tumor, responsible for more than 18% of cancers deaths in the world [1]. In Brazil, more than 30,000 new cases were diagnosed in 2020, and LC was the main cause of cancer-related death among men (13.6%); among women, it was the second-highest cause of cancer death (11.6%) [2].

Approximately 80% of patients are diagnosed with non-small cell lung cancer (NSCLC). The main subtypes of NSCLC are adenocarcinoma, squamous cell carcinoma, and large cell carcinoma, which are then categorized into heterogeneous subgroups of tumors with different therapeutic strategies. Furthermore, rapid advances in understanding the molecular pathogenesis of NSCLC have demonstrated that NSCLC is a heterogeneous group of diseases. Late diagnosis, in advanced or metastatic stages, reduces relative survival rates to about 8% [3,4,5]. In patients with NSCLC, no targetable genetic alterations, and no contraindications to immunotherapies, programmed death-1 (PD-1)/programmed death-ligand 1 (PD-L1) inhibitors, either as monotherapy or in combination, have become the standard of care. However, even in this setting, not all patients benefit due to the heterogeneity and genetic composition of the disease [6,7,8,9]. Therefore, it is necessary to implement new target therapies, as well as to identify patients who will benefit from them.

Evaluating circulating tumor cells (CTCs) derived from patients with non-small cell lung cancer (NSCLC) may contribute to identifying new therapeutic targets and potentially responding patients [10,11]. CTCs interact with other cells present in the extra-tumor environment. Very early in the bloodstream, CTCs stimulate the activation of platelets that express transforming growth factor-β (TGF-β). CTCs, as an adaptive mechanism, express TGFβ-RI (transforming growth factor-β receptor type 1), forming the platelet-CTC cluster, which protects CTCs from immune attacks mediated by Natural Killers (NK) cells, as well as from the stress of the extra-tumor environment [12,13,14].

Among the mechanisms of camouflage and escape, CTCs can express surface proteins such as cluster of differentiation 47 (CD47), which play a fundamental role in the phagocytic behavior of macrophages [15,16]. Inhibition of macrophage-mediated phagocytosis depends on signals emitted by target cells as well as macrophages. The link between the CD47 protein and the signal-regulatory protein α (SIRPα), expressed on the surface of macrophages, acts as a stop signal for phagocytosis. Its abundant expression in tumor cells works as an inhibitor signal of phagocytosis, “don’t eat me”. In this way, tumor cells acquire the ability to escape from phagocytosis, ensuring another mechanism evasion of the immune system [17,18,19,20].

In others solid tumors, the interaction of CTCs with platelets and macrophages have been studied [21,22,23,24]. However, in NSCLC, this gap remains unexplored. Here, we investigate whether CTCs and/or circulating tumor cell clusters (CTM) from NSCLC patients express CD47 and TGF-β receptor (two immune escape molecules) and their prognostic implications.

## 2. Results

The sample included metastatic NSCLC patients (*n* = 45), with baseline collection. The median age of the patients was 67 years (range = 37–77 years) and 55.6% were women. Among the participants, 20% had chronic obstructive pulmonary disease (COPD), 40% were ex-smokers, and 22.2% had a history of family history of lung cancer. During the study, 33.4% of patients (*n* = 15) died. Loss of follow-up occurred for eight patients (two died before the T2 collection and six withdrew consent before the T2 collection) (Table 1).

Adenocarcinoma was the most common subtype of NSCLC (97.8%). Bone metastases were observed in 16 patients (35.5%) at diagnosis, including patients with metastases at multiple sites. In 37.8% of the tumors, PD-L1 expression was low (<50%), and in 40%, this protein expression was not identified using immunohistochemistry reaction. Disease progression, after baseline collection (T1), was observed in 51.11% patients (*n* = 23). The more common sites of progression disease were lung (9/23 patients), bones (6/23 patients), and liver (6/23 patients), including progression at multiple sites. In Table 2, the characteristics of the disease are described. *EGFR* mutations were identified in the tumor of 16/45 patients (35.6%). Other mutated genes, as well as their specific mutations, are described in Table 3.

### 2.1. Analysis of Association of Circulating Tumor Cells and Circulating Tumor Microemboli with Progression-Free Survival

**Figure 1:**
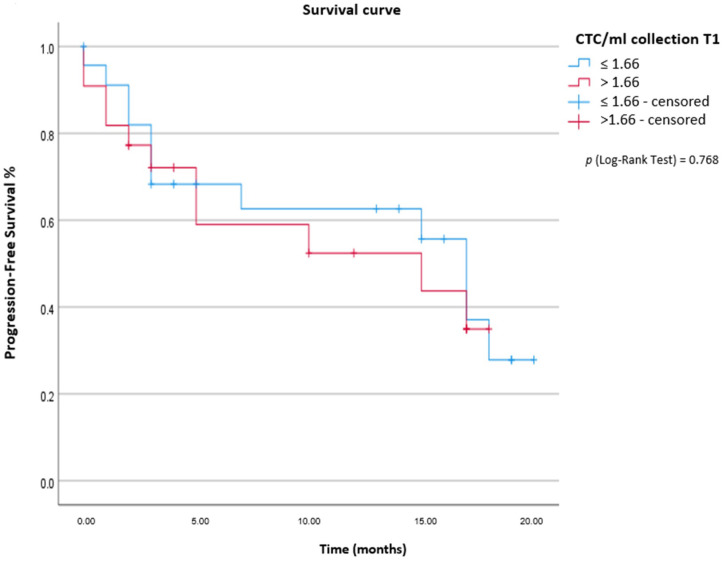
Analysis of progression-free survival (PFS) of CTCs at T1 collection determined according to the cut-off of 1.66 CTC/mL of blood.

**Figure 2:**
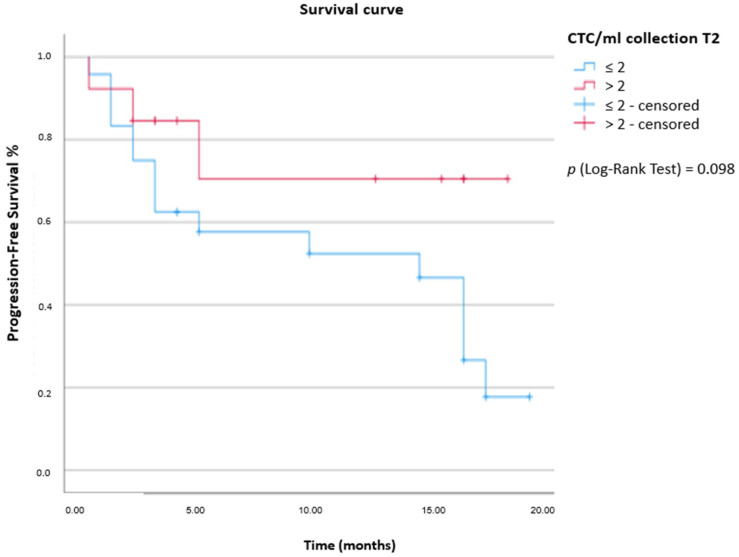
Analysis of progression-free survival (PFS) of CTCs at T2 collection determined according to the cut-off of 2 CTC/mL of blood.

**Figure 3:**
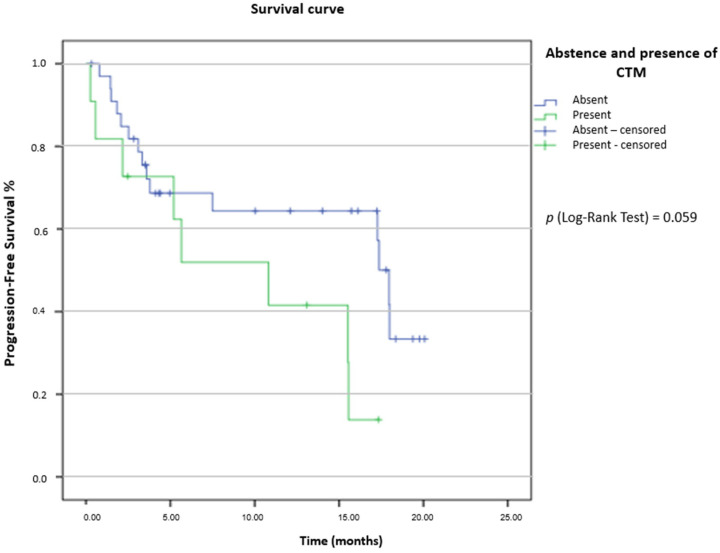
Analysis of progression-free survival (PFS) of CTM. PFS determined for patients with presence or absence of CTM independently of collection. Patients with CTM experienced poor PFS.

CTCs were detected in 82.2% (37/45 patients) At T1 collection and 94.5% (35/37 patients) at T2 collection. At T1, the mean and median of CTC/mL of blood was 2.85 and 1 CTC/mL (range: 0.33–11.33 CTCs/mL), respectively. At T2, the mean was 2.66 CTC/mL, and the median was 1.33 CTC/mL of blood (range: 0.33–9.67 CTCs/mL). We individually analyzed T1/T2 collections, concerning the volume of CTCs/mL of blood, for the PFS endpoint. For T1 collection, a cut-off of 1.66 CTC/mL of blood was established. For patients (23/45) with ≤1.66 CTC/mL of blood, the median PFS was 17 months [CI 95% 14.22–19.78] vs. 15 months for those with blood counts >1.66 CTC/mL [CI 95% 0–31.38]. There was no statistical significance between the groups [p (log-rank test) = 0.768] (Figure 1). A cut-off of 2 CTC/mL of blood was established for the PFS endpoint in the T2 collection. For patients (24/37) with ≤2 CTC/mL of blood, the median PFS was 15 months [CI 95% 6.76–23.24], and for the group (13/37 patients) with blood counts >2 CTC/mL, the PFS median was not determined. There was no statistical significance between the groups [p (log-rank test) =0.098] (Figure 2). CTMs were observed in 11/45 patients (24.5%). Using the Kaplan–Meier method, we determined a PFS median of 10 months [IC 95% 2.14–17.86] for patients who had CTMs independently of collection (T1 and/or T2) vs. 17 months [CI 95% 8.55–25.45] for the group that did not present CTMs [p (log-rank test) = 0.059] (Figure 3). Kinetic analysis between sample timepoints was performed; however, there was no statistical significance to PFS. There was a correlation between the volume of CTC/mL of blood and the presence/absence of CTM. Of the 11/45 patients with CTM, 9 patients had >1.66 CTC/mL of blood and 2 had ≤1.66 CTC/mL (of a total of 11 patients with CTM). For 21/34 patients without CTM, the counts of CTC/mL of blood was ≤1.66 CTC/mL (Fisher’s exact test: *p* = 0.014). CTM presence was statistically significant for disease progression, as 6/11 patients with CTM experienced disease progression (Fisher’s exact test: *p* = 0.017).

### 2.2. Expression of CD47 and TGFβRI in CTCs

**Figure 4:**
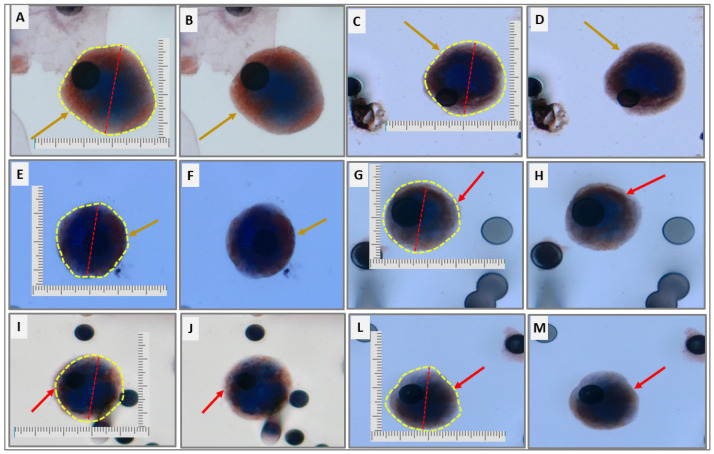
CTCs immunostaining of patients with metastatic NSCLC. (**A**–**F**) CTCs expressing CD47 antibody are indicated by yellow arrows. (**G**–**M**) Positive CTCs for TGFβRI are indicated by red arrows. CTCs were delimited by dashed yellow circle and their diameters were indicated by red dashed. All CTCs were stained using DAB chromogen. Images were taken at ×400 (**A**–**F**,**I**,**J**) and ×600 magnification (**G,H,L,M**) using a light microscope (Research System Microscope BX61—Olympus, Tokyo, Japan) coupled to a digital camera (SC100—Olympus, Tokyo, Japan). Morphological characteristics of CTCs were observed: cells with diameter more than 8 µm; hyperchromatic nucleus and cytoplasm scarcity.

Through the immunocytochemistry reaction, we verified the expression of CD47 and TGFβRI in the enriched CTCs. At T1 collection, 26/45 patients (57.78%) expressed CD47 and 25/45 (55.55%) expressed TGFβRI. At T2 collection, 21/37 patients (56.05%) with CTCs expressed CD47 and 22/37 patients (59.45%) expressed TGFβRI (Figure 4).

The PFS endpoint was determined using the Kaplan–Meier method for each marker, based on its presence or absence, in the CTCs in any sample timepoints. The PFS for patients without CD47 in CTCs at T1 collection was 17 months [CI 95% 13.9–20.63] vs. 10 months for those with its expression [CI 95% 0.00–27.26] [p (log-rank test) = 0.370]. In the T2 collection, the PFS was 15 months for patients who did not express CD47 in CTCs [CI 95% 7.95–23.17], and for patients who expressed CD47 in CTCs, the median PFS was not reached. [p (log-rank test) = 0.701]. The presence or absence of TGFβRI expression in CTCs at T1 collection did not result in a PFS difference: 15.52 months for CTCs without expression [CI 95% 1.75–29.3] vs. 15.55 months for those with TGFβRI expression in CTCs [CI 95% 7.823–23.29] [p (log-rank test) = 0.573]. At T2 collection, the absence of this marker resulted in a PFS of 15 months [CI 95% 8.38–22.73] vs. 17 months [CI 95% 0.00–38.79] [p (log-rank test) = 0.627]. So, there was no statistical significance in any sample timepoints for any marker evaluated.

The combined statistical analysis of CD47 and TGFβRI expressions At T1 showed that there is an association between the expression of these proteins, as 19/26 patients (73.1%) who expressed CD47 also expressed TGFβRI (Fisher’s exact test: *p* = 0.007) (Table 4). However, this association was not related to PFS. Kinetic analyses between sample two timepoints were performed for both markers, and no statistical significance to PFS was found.

### 2.3. Expression of CD47 and TGFβRI in CTM and PFS Analyses

**Figure 5:**
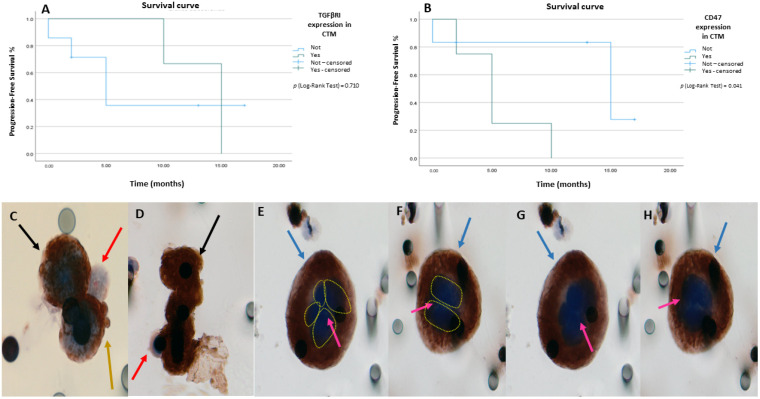
Analysis of progression-free survival (PFS) for expression of TGFβRI and CD47 in CTM. (**A**) PFS for TGFβRI expression in CTM; (**B**) PFS curve for expression of CD47 in CTM; (**C**,**D**) heterotypic CTM associated with immune cells (red arrows) and platelets (yellow arrows) expressing TGFβRI indicated by black arrows; (**E**–**H**) an example of a encapsulated CTM/giant cell found positive for CD47 antibody indicated by blue arrows. Pink arrows indicate well-defined hyperchromatic nuclei and highlighted by yellow circles observed the multiple nucleus of the giant cell/eCTM. All CTMs were stained using DAB chromogen. All images were taken at ×400 magnification using a light microscope (Research System Microscope BX61—Olympus, Tokyo, Japan) coupled to a digital camera (SC100—Olympus, Tokyo, Japan).

Through immunocytochemistry analysis, we observed CTMs expressing TGFβRI in 3/11 patients (27.27%). For this group, there was no statistical significance for PFS endpoint [p (log-rank test) = 0.710] (Figure 5A). CTMs expressing CD47 were observed in 4/11 patients (36.37%). The median PFS for CTM CD47+ was 5 months [CI 95% 2.45–7.54] vs. 15 months for patients who did not express CD47 [CI 95% 2.7–27.29] [p (log-rank test) = 0.041] (Figure 5B). CTMs expressing TGFβRI and CD47 can be observed in Figure 5C–H.

## 3. Discussion

In this study, we focused on the prognostic potential of CTCs from NSCLC patients. Using the ISET Technology^®^, we identified, in the initial and follow-up collections, CTCs in 82.2% and 94.5% of patients, respectively, and CTMs were observed in 24.5% of patients. ISET was previously tested in the NSCLC scenario, and, as in our work, high rates of CTCs detection were observed [25,26,27].

As far as we know, this is the first study that has analyzed the expression of CD47 in CTCs and CTMs of patients with NSCLC. The expression of CD47 protein, which acts as an immune checkpoint, has been studied in some solid tumors. In a study with breast cancer patients, Papadaki [22] demonstrated that CTCs CD47+ can be found in initial, recurrent, and metastatic disease (*p* = 0.036), being more frequently observed in patients with recurrent and metastatic disease (*p* = 0.009). In addition, they observed that, when highly expressed in CTCs, CD47 was associated with disease progression (*p* = 0.005) and with worse PFS (*p* = 0.010). There is also a clinical study evaluating CD47 (don’t eat me) blockade as immunotherapy, in association with PD-L1 blockade (don’t find me) [28].

In our study, we observed that patients with CTMs CD47+ had worse PFS when compared with patients who did not express this protein (*p* = 0.041). For patients with CTCs CD47+, there was no statistical significance between marker expression and PFS. In a study conducted by Arrieta [29], the expression of CD47 in the tumor tissue of patients with NSCLC was not statistically significant for PFS. Giatromanolaki [30] investigated the expression of CD47 in 98 patients with NSCLC. A total of 29/98 patients (29.6%) expressed some level of CD47 in the tumor tissue; however, it was not possible to associate the expression with disease-specific overall survival. In our study, we observed that 26/45 patients (57.78%) had CD47+ CTCs at T1 collection, and at T2 collection, 21/37 patients (56.05%) had CD47 expression in CTCs. Although we did not find statistical significance in our results, the more expressive finding of CD47 in circulating cells may suggest that this marker can bring more information about the tumor dynamics and immune cell interaction when analyzed in tumor circulating components.

We also evaluated the expression of TGFβRI, which is involved in the regulation of migration, differentiation, and cell proliferation in different cell types. Previously, our group has identified that this marker, expressed in CTCs, was a worse prognostic factor for patients with head and neck cancer [21]; however, it was not possible to establish the same premise in patients with NSCLC.

We analyzed the association between CD47 and TGFβRI expression in CTCs. The combined statistical analysis of the markers showed that there was an association between these two molecules. In our study, 19/26 patients (73.1%) expressed both proteins. Hendriks [31] and colleagues demonstrated that bispecific blocking of CD47 and EGFR by the BsAb CD47xEGFR-IgG1 antibody increased the selective phagocytosis of CD47+ and EGFR+ cells, avoiding the nonspecific blocking of CD47, since this protein is expressed in different cells. Here, we observed that there was an association between CD47 and TGFβRI, both being used by tumor cells for evasion of the immune system. This understanding may contribute to new studies with double immune blockage.

For metastases to occur, it is necessary that the metastatic sites present a microenvironment like that of the NSCLC: highly vascularized to maintain tumor oxygenation, with cytokines and immune cells resulting from exposure to oncogenic factors such as exposure to tobacco. The respiratory tract presents its peculiarities regarding the immune response. It counts with the presence of alveolar macrophages capable of phagocyting inhaling substances and pathogens, modulation of cytokines and chemokines (IL-1β, IL-6, -7, -8, TNF-α), adhesion molecules, and other substances only present in the lung, such as surfactants proteins. When tumor cells leak into the bloodstream, they are exposed to immune recognition actions different of those of their niche, and shear stress. Even with adaptative mechanisms to resist attacks, it is necessary to find the ideal place to colonize [32,33].

We also correlated the presence of CTMs with PFS, regardless of their protein expression. It is well known that the presence of CTMs is a poor prognosis factor in several types of solid tumors such as breast, prostate, colorectal, and renal [34,35,36,37]. Our results showed that the presence of CTMs had worse median PFS: 10 months vs. 17 months for those who do not have clusters of CTCs (*p* = 0.05). Recent studies have shown that, for patients with early stage lung cancer with CTMs, there is a tendency towards worse PFS [38]; however, for patients with NSCLC in stages III and IV, the presence of CTMs correlated, in fact, with worse PFS [37], corroborating with our findings.

Although we have observed encouraging results, there are some weak points that need to be highlighted, such as the small sample size and the T2 collection during treatment. These points need to be adjusted in future studies. In addition, this was a pilot study that showed the association of CTCs and CTMs with the expression of immune evasion markers, as well as with disease progression and poor PFS. Due to the performance of several statistical analyses with small sample numbers, it is worth mentioning that some of them may result in false-positive analyses.

## 4. Materials and Methods

**Figure 6:**
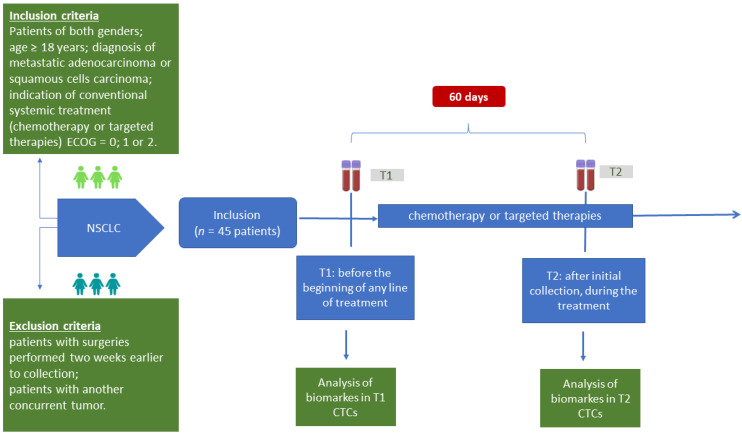
Study design. Flowchart showing the number of patients included and collections performed in the project. T1: before the beginning of any line of treatment; T2: 60 days after initial collection, during the treatment.

This prospective study was conducted at A.C. Camargo Cancer Center, located in São Paulo, Brazil (institutional review board no 2496/18C). Metastatic NSCLC patients with diagnosis of adenocarcinoma or squamous cell carcinoma confirmed using anatomopathological examination, of both genders, with ECOG performance status equal to 0, 1, or 2, able to receive conventional systemic treatment (chemotherapy or targeted therapies), were invited to participate and included after signing the consent agreement. Patients with surgeries performed two weeks prior to collection or with another concurrent tumor were not eligible for participation. Peripheral blood samples (10 mL) were collected from patients (*n* = 45) at two different times to analyze the CTCs dynamically: T1—before the beginning of any line of treatment, and T2—60 days after initial collection, during the treatment (Figure 6).

### 4.1. Enrichment of Circulating Tumor Cells Using ISET^®^

Blood samples were collected in EDTA tubes (BD Vacutainer^®^, BD, Belo Horizonte, MG, Brazil) and preserved with delicate constant agitation until processing. Samples were filtered in ISET^®^ (Isolation by SizE of Tumors cells, Rarecells, France) according to manufacturer’s instructions. Blood was diluted in up to 80 mL of lysis buffer containing 0.02% formaldehyde and incubated for 10 min in room temperature. Lysed samples were filtered through a polycarbonate membrane composed of spots with 8 µm diameter micropores. Then, the membrane was washed once with phosphate-buffered saline (PBS). To preserve cell integrity, the filtration pressure was optimized to −10 kPa. After processing, filters were dried, wrapped in an aluminum sheet, and stored frozen at −20 °C until use.

### 4.2. Immunocytochemistry

To analyze TGFβRI and CD47 in CTCs and CTMs, we performed dual immunocytochemistry (ICC), combining each antibody with anti-CD45 for leukocyte cell exclusion. We put ISET membrane’s spots in a 24-well plate. In each well, to perform antigenic retrieval, 1 mL of Retrieval Solution (EnVision FLEX Dako ^TM^, low pH, Dako^TM^, Santa Clara, CA, USA) was added and heated (three repetitions of 1 h 40 min heating in a microwave oven), followed by hydration with tris-buffered saline (TBS), dilution 1:10. The cells were permeabilized with TBS 0.2% + Triton X-100 for 5 min at room temperature and incubated with Endogenous Peroxidade Blocked (EnVision FLEX—Dako^TM^), for 5 min in the dark, for enzymatic blockade. After each step, the spots were washed once with TBS. The spots were incubated overnight with antibodies diluted in TBS 10% fetal serum, followed by incubation with Horseradish Peroxidase—HRP (EnVision FLEX—Dako^TM^) for 20 min. The revelation was made using chromogen Diaminobenzidine 3.3’ (DAB) (Dako^TM^), incubated for 10 min. Before adding the second antibody, the cells were incubated with sulfuric acid (0.1 M) to prevent possible non-specific reactions of the HRP molecules with the second chromogen. After incubating the second antibody for two hours, we performed a new incubation with HRP for 20 min. The revelation was made using Magenta chromogen (EnVision FLEX—Dako^TM^) incubated for 5 min (after each step, the spots were washed once with TBS), followed by hematoxylin staining for 2 min. Then, the spots were washed three times with distilled water. Mounting Medium—Dako was used to bond the spots in glass microscope slide. The slides were read in a Research System Microscope BX61—Olympus, attached to a digital camera (SC100—Olympus, Tokyo, Japan). The cytopathological characterization of CTCs was performed according to the malignant criteria: nuclear size equal or greater than 16μm, irregularity of the nuclear border, visible presence of cytoplasm, and high nucleus-cytoplasm ratio (>80%) [39]. CTCs were quantified using spot analyzed, each spot of the ISET membrane corresponding to 1 mL of blood [40]. CTCs in clusters of 2 or more cells were considered CTMs [41]

### 4.3. Immunocytochemistry Control

Before ICC in the ISET spots from patients, we performed positive and negative controls for each antibody. We used cell lines spiked in healthy blood. The cells were chosen as they expressed or did not express the markers of interest as described in The Human Protein Atlas (http://www.proteinatlas.org/).

**Figure 7:**
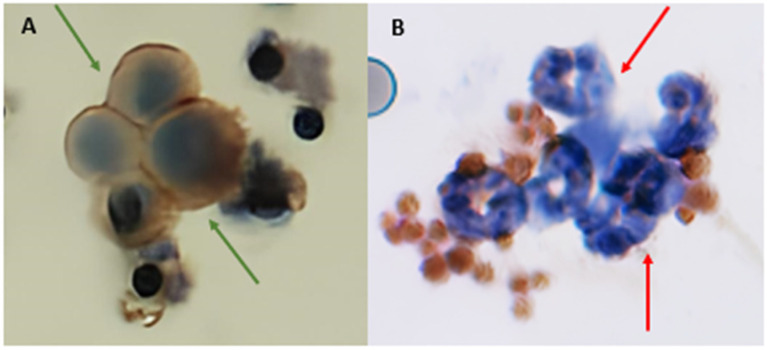
Positive and negative CD47 controls. (**A**) SKOV3 line cell spiked in healthy blood, stained with CD47 antibody, indicated by green arrows, revealed by DAB chromogen. (**B**) Neutrophils from NSCLC patients were used as a negative control, indicated by red arrows, visualized with hematoxylin. Some platelets are stained with DAB (unspecific staining). Images were taken at ×400 (**A**) and ×600 (**B**) magnification using a light microscope (Research System Microscope BX61—Olympus, Tokyo, Japan) coupled to a digital camera (SC100—Olympus, Tokyo, Japan).

Two antibodies were used: CD47 and TGFβR1. CD47 Abcam (Abcam, Waltham, MA, USA) (ab236234) dilution 1: 300 was standardized using the SKOV3 line cell as the positive control. The CD47 negative control was the neutrophils of patients with NSCLC. Although several molecules are redundant in many cells, CD47-SIRPα seems to be specific, as CD47 is expressed in healthy and cancer cells and SIRPα only in myeloid cells (Figure 7) [42]. TGFβR1 Merck (abf 17-1) dilution 1: 500 was previously standardized by our group in a positive control A549 cell line. The negative control was an MCF-7 cell line spiked with healthy blood [43]. The antibody CD45 (ab10559) was used for leukocyte exclusion; its standardization was performed previously by our group [43], using leukocytes as the positive control and the cell line A549 as the negative control.

### 4.4. PD-L1 Expression and Genes Mutated in the Primary Tumor

Expression levels of PD-L1 in tumor tissue, as well as gene mutations, were obtained through medical records.

### 4.5. Statistical Analysis

Patient characteristics analyses were reported as relative/absolute frequencies for qualitative variables. For the outcome progression-free survival (PFS), the period analyzed was the date of initial blood collection (T1) until the date of progression. Progressive disease (PD) was defined according to RECIST 1.0, which describes as progression at least a 20% increase in the sum of diameters of target lesions or evidence of new metastatic lesions on follow-up images.

The analyses were performed using Kaplan–Meier curves and log-rank tests. To establish the cut-off, we used the maximally selected rank statistics method proposed by Lausen [44] (based on the value of the log-rank statistic). This method evaluates all possible cut-off points, considering the follow-up time for the time/event results. Thus, it is possible to maximize the difference between the survival curves because there is no overlap of the censored events. All analyses were performed using SPSS software version 28. The level of significance was 5% for all tests.

## 5. Conclusions

In this study, we isolated, quantified, and analyzed CTCs in 82.2% of patients at T1 collection and in 94.5% at T2 collection. CTMs were observed in 24.44% of the patients included. CTCs and CTMs were statistically associated with disease progression and reduction of PFS. We also demonstrated that CTCs and CTMs derived from NSCLC patients express the markers of immune evasion TGFβRI and CD47. In addition, we showed the expression of those proteins at the same time in 73.1% of the enriched CTCs, indicating that these cells were using both immune evasion mechanisms.

This was the first study to investigate the expression of CD47 in CTCs and CTMs of patients with metastatic NSCLC and related the expression with PFS. We showed that CD47 expression in CTMs was associated with worse PFS.

## Figures and Tables

**Table 1:** Characteristics of NSCLC patients included.

Patients Characteristics	*N*	%
Gender		
Male	20	44.4
Female	25	55.6
Total	45	100.0
Median age—67 years		
≤67	23	51.2
>67	22	48.8
Total	45	100.0
COPD history		
Yes	9	20.0
No	28	62.2
Not specified	8	17.8
Total	45	100.0
Smoking history		
Non-smoking	21	46.7
Ex-smoker	18	40.0
Smoker	4	8.9
Not specified	2	4.4
Total	45	100.0
Family history of lung cancer		
Yes	10	22.2
No	35	77.8
Total	45	100.0
Death after study inclusion		
Yes	15	33.4
No	30	66.6
Total	45	100.0

Abbreviation: COPD: chronic obstructive pulmonary disease.

**Table 2:** Characteristics of primary tumors of patients with NSCLC included.

Disease Characteristics *	*N*	%
Histological grades		
Indeterminated	38	84.4
Moderately differentiated	2	4.4
Poorly differentiated	5	11.2
Total	45	100.0
Histological types		
Adenocarcinoma	44	97.78
Squamous cell carcinoma	1	2.2
Total	45	100.0
Metastatic sites at diagnosis		
Central nervous system	2	4.4
Bones	8	17.9
Lung	1	2.2
Pleural	1	2.2
Hepatic	2	4.4
Lymph nodes	2	4.4
Adrenal	1	2.2
2 sites **	8	17.9
3 sites **	7	15.6
4 sites **	3	6.6
Others	4	8.9
Undetermined	6	13.3
Total	45	100.0
PD-L1 expression score (%) in primary tumor		
≥50	10	22.2
1–49	17	37.8
<1	18	40.0
Total	45	100.0
Treatments		
ICI	8	17.8
TKI	9	20.0
ICI + CT	12	26.7
CT	13	28.9
Not specified	3	6.6
Total	45	100.0
Disease Progression after enrollment		
Yes	23	48.9
No	22	51.1
total	45	100.0
Progression sites		
Lung	4	17.4
Central nervous system	4	17.4
Bones	2	8.7
Lymph nodes	1	4.3
2 sites ***	5	21.8
3 sites ***	1	4.3
4 sites ***	2	8.7
Others	4	17.4
Total	23	100.0

Abbreviations: AR: androgen receptor; CT: chemotherapy; CNS: central nervous system; ICI: immune checkpoint inhibitors; IDH1: gene isocitrate dehydrogenase (NADP (+)); AR: androgen receptor; TKI—tyrosine kinase inhibitors; Observations: * medical records. ** two sites: lymph nodes + bones; CNS + bones; pleural + peritoneum; bones + pleural; lymph nodes + pleural; pleural + CNS; bones + lung; three sites: CNS + bones + hepatic; bones + hepatic + adrenal; lung + CNS + peritoneum; bones + pleural + hepatic; lung + pleural + hepatic; lymph nodes + bones + hepatic; four sites: lung + CNS + lymph nodes + pleural; lung + CNS + lymph nodes + bones; lymph nodes + bones + pleural + adrenal. *** two sites: hepatic + bones; hepatic + mediastinum; lung + mediastinum; lung + hepatic; three sites: bones + lymph nodes + soft tissue; four sites: lung + hepatic + bones + CNS; lung + hepatic + bones + lymph nodes.

**Table 3:** Mutated genes in primary tumor.

Mutated Genes	Mutations	*N*	%
*EGFR*	NM_005228.5:c.2235_2249del p.(Glu746_Ala750del)	4	8.9
*EGFR*	NM_005228.5:c.2239_2253delinsCCT p.(Leu747_Thr751delinsPro)	1	2.2
*EGFR*	NM_005228.5:c.2239T>G p.(Leu747Val) NM_005228.5:c.2582T>A p.(Leu861Gln)	1	2.2
*EGFR*	NM_005228.5:c.2236_2250del p.(Glu746_Ala750del); NM_005228.5:c.2369C>T p.(Thr790Met)	1	2.2
*EGFR*	NM_005228.5:c.2240_2254del p.(Leu747_Thr751del)	1	2.2
*EGFR*	NM_005228.5:c.2239_2251delinsC p.(Leu747_Thr751delinsPro)	1	2.2
*EGFR*	NM_005228.5:c.2235_2249del p.(Glu746_Ala750del); NM_005228.5:c.2369C>T p.(Thr790Met)	2	4.4
*EGFR*	NM_005228.5:c.2239_2248delinsC; p.(Leu747_Ala750delinsPro)	1	2.2
*EGFR*	NM_005228.5:c.2240_2257del18 p.(Leu747_Pro753delinsSer)	1	2.2
*EGFR*	NM_005228.5:c.2573T>G p.(Leu858Arg)	1	2.2
*EGFR; MUTYH; TP53*	NM_005228.5:c.2309_2314dup p.(Asn771_Pro772insHisAsn); NM_001048174.2:c.1186_1187insGG p.(Leu396fs); NM_000546.6:c.783-2A>T	1	2.2
*EGFR; CDK4*	NM_005228.5:c.2237_2251del p.(Glu746_Thr751delinsAla); amplification	1	2.2
*KRAS*	NM_004985.5:c.35G>A p.(Gly12Asp)	3	6.7
*KRAS*	NM_004985.5:c.34G>T p.(Gly12Cys)	1	2.2
*KRAS; IDH1*	NM_004985.5:c.35G>T p.(Gly12Val); NM_005896.4:c.395G>T p.(Arg132Leu)	1	2.2
*KRAS; RBM10*	NM_004985.5:c.35G>C p.(Gly12Ala); NM_005676.5:c.1831dup p.(Glu611fs)	1	2.2
*KRAS; JAK3; FH; TP53; TP53; RBM10; MET; CDK4; HGF; ARFRP1; CCNE1; KDM5A*	NM_004985.5:c.35G>T p.(Gly12Val); NM_000215.4:c.2842C>T p.(Arg948Cys); NM_000143.4:c.1303G>A p.(Val435Met); NM_000546.6:c.514G>T p.(Val172Phe); NM_000546.6:c.511G>T p.(Glu171*); NM_005676.5:c.2485G>T p.(Glu829*); amplification; amplification; amplification; amplification; amplification; amplification	1	2.2
*KRAS; PIK3CA*	NM_004985.5:c.35G>A p.(Gly12Asp); NM_006218.4:c.1624G>A p.(Glu542Lys);	1	2.2
*KRAS; AR*	NM_004985.5:c.35G>T p.(Gly12Val); amplification	1	2.2
*MET*	NM_000245.4:C.3028+3A>T; exon 14 skipping [MET(13)-MET(15)]	1	2.2
*MET; NFKBIA; NKX2-1*	amplification	1	2.2
*MET; MYC*	NM_000245.4:c.3028+1G>A; amplification	1	2.2
*NRAS*	NM_002524.5:c.182A>T p.(Gln61Leu)	1	2.2
*HRAS*	NM_005343.4:c.37_38delinsTT p.(Gly13Phe)	1	2.2
*ARID1A; KEAP1; TP53; TP53; MET; RICTOR; EPHB4; MCL1*	NM_006015.6:c.5407G>T p.(Glu1803*); NM_203500.2:c.1426G>T p.(Gly476Trp); NM_000546.6:c.920-1G>T; NM_000546.6:c.254del p.(Pro85fs); amplification; amplification; amplification; amplification	1	2.2
*BRAF; MTOR; AXL; SETD2*	NM_004333.6:c.1799T>A p.(Val600Glu); NM_004958.4:c.5395G>A p.(Glu1799Lys); NM_021913.5:c.883C>T p.(Arg295Trp); NM_014159.7:c.1177C>T p.(Arg393*)	1	2.2
Not available		8	17.8
Not detected		5	11.1
Total		45	100.0

**Table 4:** Association of CD47 and TGFβRI expression in CTCs derived from NSCLC patients.

	CD47 Expression—T1			
Expression	Category	Absence *n* (%)	Present *n* (%)	*p* Value
TGFβRI—T1	No Yes Total	13 (68.4) 6 (31.6) 19 (100.0)	7 (26.9) 19 (73.1) 26 (100.0)	0.007

## Data Availability

Not applicable.
